# The impact of internet addiction on non-suicidal self-injury among adolescents: a moderated chain mediation model

**DOI:** 10.3389/fpsyg.2025.1735137

**Published:** 2026-01-14

**Authors:** Changjiao Wei, Xiaofei Dong

**Affiliations:** 1 School of Teacher Education, Daqing Normal University, Daqing, China; 2 School of Psychology, Zhejiang Normal University, Jinhua, China; 3 Key Laboratory of Intelligent Education Technology and Application of Zhejiang Province, Zhejiang Normal University, Jinhua, China

**Keywords:** depression, internet addiction, non-suicidal self-injury, social anxiety, trait meta-mood

## Abstract

**Objective:**

Adolescence is a critical period for personality development and a high-risk phase for psychological conflicts. With the increasing severity of mental health issues among adolescents, this study investigates the mechanisms underlying the relationship between internet addiction (IA) and non-suicidal self-injury (NSSI) in adolescents, focusing on the mediating roles of social anxiety and depression and the moderating effect of trait meta-mood. The aim of this study is to uncover the psychological motivations behind NSSI and provide a theoretical basis for identifying potential risks to adolescent mental health, ultimately contributing to the prevention of psychological crises.

**Methods:**

A total of 692 high school students from Heilongjiang Province, China, completed the Internet Addiction Test (IAT), the Deliberate Self-Harm Inventory (DSHI), the Liebowitz Social Anxiety Scale (LSAS), the Beck Depression Inventory-II (BDI-II), and the Trait Meta-Mood Scale (TMMS). Structural equation modeling was used to examine the mediating and moderating effects among the variables.

**Results:**

(1) A significant positive correlation was found between internet addiction and NSSI (*r* = 0.278, *p* < 0.01). (2) Social anxiety (indirect effect = 0.042) and depression (indirect effect = 0.019) formed a chain mediation pathway, explaining 26% of the total effect. (3) Trait meta-mood moderated the first half of the mediation pathway (*R*^2^ = 0.161, *p* < 0.001), with the mediating effect of internet addiction being stronger at high levels of trait meta-mood (0.08) than at low levels (0.036).

**Conclusion:**

Internet addiction exacerbates the risk of NSSI in adolescents through a progressive pathway of “social anxiety → depression.” trait meta-mood plays a crucial role in moderating this process, highlighting the importance of emotional regulation in mitigating the adverse effects of internet addiction on adolescent mental health.

## Introduction

Non-suicidal self-injury (NSSI) refers to the deliberate and repeated act of intentionally harming one’s own body without suicidal intent, such as cutting, scratching, burning, scalding, or biting (Lloyd-Richardson et al., 2007). Some scholars also include indirect forms of self-harm beyond direct physical injury. In the past, owing to insufficient societal and medical understanding of this behavior and its often concealed nature, it was not considered a serious mental health issue. However, despite its long-standing lack of attention, recent epidemiological data have revealed its severity: NSSI is a highly prevalent behavior, with the risk significantly increasing during adolescence and gradually decreasing with age. Research in the field of cognitive neuroscience indicates that NSSI can become chronic through self-reinforcing mechanisms and is closely linked to psychiatric disorders. These studies have also elucidated the potential mechanisms underlying the development of chronic self-injury, suggesting that this behavior may become fixed over time owing to its self-reinforcing nature. The reduction in aversion to physical pain and the enhancement of self-schema related to NSSI make its occurrence more likely (Liu, 2017). The latest edition of the Diagnostic and Statistical Manual of Mental Disorders (DSM-5) includes self-injury as a diagnostic criterion for borderline personality disorder, and the World Health Organization’s International Classification of Diseases has also introduced NSSI as a distinct condition. NSSI is a common behavior among patients with bipolar disorder, and abnormalities in serum metabolites in these patients may be linked to its pathological mechanisms, with these biomarkers being significantly associated with the occurrence of NSSI (American Psychiatric Association, 2013). However, research on adolescent NSSI varies across different cultural, economic, and social contexts, as do differences in diagnostic criteria and data collection methods (Patton et al., 2009). A review of adolescent self-injury research revealed that methods using self-injury checklists provide higher prevalence estimates than single-item questions do (Lim et al., 2019). This has led to highly heterogeneous prevalence estimates of NSSI in non-clinical samples, raising concerns about their reliability and hindering broader exploration of NSSI (Muehlenkamp et al., 2012). As a public health issue, there is an urgent need to establish reliable prevalence rates for NSSI (Swannell et al., 2014). Related research has prognostic significance for adolescent personality disorders, providing a scientific basis for early identification and treatment and reducing the risk of widespread and disabling psychological illnesses and behavioral disorders (Wilkinson, 2013).

In addition, NSSI is also associated with significant symptoms of other psychiatric disorders, such as depression, anxiety disorders, obsessive-compulsive disorder, and anorexia nervosa (Gámez-Guadix et al., 2022). The risks posed by adolescent NSSI are not confined to the teenage years; they also predict later suicide attempts in young adulthood (Fergusson et al., 2000). Suicide and non-suicidal self-injury are overlapping behaviors, and self-injury is a strong risk factor for suicide attempts. Compared with preinjury levels, mixed effects models indicate a significant increase in suicidal ideation following an episode of self-injury (Wilkinson et al., 2011). Therefore, the early detection and treatment of NSSI during adolescence are of lifelong importance for individual development (Herzog et al., 2022). Research on NSSI during this stage helps us understand the underlying psychological, social, and biological factors, thereby contributing to the overall improvement of mental health in society.

## Literature review

### Internet addiction and adolescent non-suicidal self-injury

Adolescents often express their inner pain and struggles through self-injury, and they may share their experiences of NSSI online, which further reinforces this behavior and makes the internet a habitual outlet or coping mechanism for them. Online content and communities related to self-injury can exacerbate their self-injury behaviors. Internet activities facilitate the dissemination of self-injury information among potential at-risk groups and may connect individuals with self-injury tendencies across schools or communities (Marchant et al., 2017). From the perspective of social cognitive theory, adolescents can learn behaviors associated with NSSI through social interactions and online information exchange while also seeking support and validation (Liu et al., 2022). Davis’s cognitive–behavioral model posits that internet addiction (IA) stems from cognitive distortions in online activities and the reinforcement of internet use, with maladaptive cognition being the primary source of aberrant behavior (Davis, 2001).

These theoretical mechanisms are supported by empirical research; for example, longitudinal studies have shown that individuals with moderate to severe internet addiction have a fivefold increased risk of self-harm compared to normal users over a six-month period (Lam et al., 2009). Case–control studies also confirmed that internet addiction is a predictive factor for increased NSSI among adolescents (Zhu et al., 2022). However, the relationship between internet addiction and NSSI remains controversial, potentially due to heterogeneity in study populations (e.g., age, comorbid mental disorders), leading to inconsistent conclusions (Tang et al., 2020). Exploring the adverse consequences and related factors of these behaviors in adolescents can help clarify their internal and external relationships and elucidate their underlying mechanisms.

Although the association between internet addiction and NSSI has been partially confirmed, the heterogeneity in the research findings suggests the need to further explore potential mediating variables. Numerous studies have demonstrated that depression, anxiety, and interpersonal problems consistently accompany the occurrence of NSSI. Depression and social anxiety are antecedents of problematic internet use, and both are present in the context of internet addiction and NSSI (Wu et al., 2022). The I-PACE model (Interaction of Person-Affect-Cognition-Execution) emphasizes that internet addiction results from the interplay of personal traits (e.g., impulsivity), affective responses (e.g., depression), cognitive biases (e.g., overly optimistic evaluations of internet benefits), and deficits in executive control. These factors serve as predisposing, moderating, or mediating variables leading to specific internet-use disorders (Brand et al., 2016). Therefore, when examining the relationship between internet addiction and adolescent NSSI, we considered factors such as depressive mood, affective responses, social anxiety, and trait meta-mood and employed a conditional process model to analyze the specific pathways through which internet addiction influences NSSI behavior.

### Mediating role of social anxiety and depression

#### Mediating role of social anxiety

Social anxiety (SA) was initially understood as the experience of fear, distress, and discomfort in public settings, accompanied by concerns about negative evaluations from others. Later definitions emphasize the intense fear and anxiety that individuals feel in social situations (Watson and Friend, 1969). The American Psychiatric Association describes it as a marked and persistent fear of social or public activities, diagnosing and treating it as a psychological disorder (American Psychiatric Association, 2013). Individuals with social anxiety who engage in online gaming establish various social relationships in virtual spaces without worrying about others’ judgments, making the avoidance domain of social anxiety the strongest predictor of the severity of internet addiction (Yücel and Üzer, 2018). The compensatory internet use model suggests that online interactions help individuals escape negative emotions and concerns about others’ evaluations, reducing social anxiety. This is particularly relevant for adolescents in the self-exploration stage, who oscillate between self-evaluations and others’ evaluations and seek to make an impact in multiple domains (Branje, 2022). The internet provides them with a freer space for self-expression, alleviating the discomfort of face-to-face interactions. Consequently, adolescents experiencing such emotional issues tend to spend more time online. Internet addiction deprives individuals of opportunities to practice social skills in real life, exacerbating symptoms of social anxiety and potentially creating a vicious cycle. In real-life settings, individuals with social anxiety continue to doubt their social competence and self-worth, leading to negative emotions, self-loathing, and even self-injury. Negative life events and emotional symptoms are risk factors for NSSI. When experiencing anxiety, distress, and fear in interpersonal interactions, individuals are more likely to resort to self-harm as a form of relief (Jiang et al., 2022). Individuals with generalized anxiety disorder and social phobia are more likely to engage in repeated self-harm, including at least one suicide attempt (Chartrand et al., 2012), making early psychological intervention and treatment particularly crucial.

#### The mediating role of depression

Depression is closely related to psychological behaviors such as internet addiction and self-injury. Internet addiction can influence an individual’s depressive state. Psychological and medical research on adolescents has shown that excessive internet use triggers emotional arousal and disrupts the transition between the prefrontal cortex and the limbic system, affecting emotional regulation and behavioral performance (Steinberg, 2005). Internet addiction can impair dopamine transmission by reducing the expression of dopamine transporters in the striatum, thereby increasing the risk of depressive symptoms (Pizzagalli et al., 2019). Individuals with depressive tendencies exhibit significantly greater risks of both internet addiction and self-injury behaviors, and the strength of their association changes with fluctuations in depression levels. When explained through emotional disorder theory, this theory attributes adolescents’ antisocial behaviors (e.g., self-injury) to abnormalities in central nervous system (CNS) circuits, which are responsible for regulating negative emotions and processing environmental cues (Zhang and Zhang, 2023). These negative emotions include depression. Therefore, we propose that self-injury behaviors and depressive emotions share a common physiological basis. Depressed individuals may exhibit various forms of internalized and externalized aggression, such as self-injury and impulsive behaviors. Depression and NSSI may share highly prevalent psychological mechanisms (Tilton-Weaver et al., 2019). Internet addiction influences NSSI through depression, and depression, as an emotional state, serves as a mediating factor in the relationship between internet addiction and NSSI.

#### The chain-mediating role of social anxiety and depression

Baker et al. (2004) on the basis of recent theories and research, proposed the negative reinforcement emotion processing model, which posits that negative emotions are the dominant motivation for maintaining addictive and problematic behaviors and play a critical role in both the initiation and relapse of such behaviors. Social anxiety and depression are closely related, with both disorders sharing mechanisms of cognitive rigidity that influence an individual’s emotions and behaviors (Rahamim et al., 2023). Within this framework, depression and anxiety, as common negative emotions, are highly comorbid with addiction and other problematic behaviors, which to some extent supports the mediating role of social anxiety and depression in the relationship between internet addiction and NSSI. Individuals with social anxiety are prone to negative self-evaluations during online interactions, which can be conceptualized as a stepping stone from one disorder to another, increasing the likelihood of secondary behavioral problems such as depression (Nordahl et al., 2018). Cross-national comparative studies have also confirmed the influence of depression and social anxiety (Lai et al., 2015). Longitudinal studies have revealed significant associations between social anxiety, depression, and suicidal ideation among adolescent populations, with the three potentially forming a vicious cycle. Social anxiety may be a precursor to depression, and the coexistence of social anxiety and depression significantly increases the risk of self-injury (Chiu et al., 2025). The internet provides adolescents with interpersonal issues, such as social anxiety, a platform to present their ideal selves, alleviating their dissatisfaction in the real world. However, this psychological compensation does not alter the behaviors induced by anxiety and depression, which provides strong support for further exploration of the chain mediating mechanisms of social anxiety and depression.

### The moderating role of trait meta-mood

Trait meta-mood (TMM), which reflects an individual’s stability and tendencies in emotional processing, encompasses the ability to attend to and perceive emotions, the degree of understanding of emotional states, and the capacity for emotional regulation (Aliya et al., 2014). As a manifestation of personality traits, the formation and development of trait meta-mood are inseparable from cognitive involvement and the interaction between emotions and cognition. Social cognitive theory (SCT) provides a relevant framework, positing that the external environment, individual internal factors, and behavioral factors are not isolated but interact with one another (Bandura, 1986). This theory helps explain the significant role of trait meta-mood in shaping individual psychological and behavioral development, as well as its influence on internet addiction, social anxiety, and NSSI. Research based on SCT suggests that trait meta-mood, as an internal variable, impacts addictive behaviors and interpersonal interactions through its effects on emotional attention and understanding of emotional states. Studies grounded in this theory have confirmed that individual cognitive factors and social interactions (environmental factors) are related to adolescent internet addiction (behavioral factors) (Yang, 2020). Self-cognitive factors, including those related to trait meta-mood, can influence adolescent social anxiety through environmental factors. Individuals who spend more time on social networks (environmental factors) also report greater addiction tendencies (behavioral factors) and psychological issues (internal individual factors) (Wu et al., 2013). Attention to emotional perception and interpersonal relationships within trait meta-mood may be important influencing factors in internet addiction. Furthermore, from the perspective of self-regulation theory, both internet addiction and NSSI are associated with an individual’s emotional self-regulation. When tracing the trajectory of internet use problems, emotional issues are often identified as underlying vulnerability factors affecting addictive behaviors (Pettorruso et al., 2020). For adolescents, when internet addiction involves varying degrees of engagement, different levels of Trait meta-mood moderate their anxiety levels. Differences in trait meta-mood levels result in varying impacts of internet addiction on adolescents, and the degree of social anxiety changes during the process of emotional perception and processing. However, the specific mechanisms of this moderating role remain underexplored. The role of trait meta-mood in the relationship between adolescent behavioral and mental health outcomes urgently requires further investigation. These studies will contribute to a better understanding of the role of trait meta-mood in adolescent mental health and provide new insights for the prevention and intervention of self-injury behaviors.

### The present study

Although some existing studies have revealed an association between internet addiction and NSSI, the underlying mechanisms remain unclear. Some studies have failed to find significant associations, suggesting that factors such as participant characteristics and emotional states may influence the consistency of results (He et al., 2024). The mediating roles of social anxiety and depression are affected by their relationship, yet the strength and direction of this relationship vary across studies (Cohen et al., 2017). Some research has identified a bidirectional association between social anxiety and depressive symptoms (Belmans et al., 2019), and the differences in findings may lead to ambiguity in understanding the roles of social anxiety and depression in development. Additionally, while research on the moderating variable of trait meta-mood is growing, its relationship with other variables (e.g., NSSI, social anxiety, and depression) has not been sufficiently explored. Trait meta-mood significantly moderates individual emotions (Fernández-Berrocal and Extremera, 2008); however, in other contexts, this moderating effect is influenced by various factors (Yildirim et al., 2023). This suggests that the mechanisms of trait meta-mood may vary depending on the research context, leading to differing interpretations of its role in mental health. The inconsistencies in these aspects of research, combined with the lack of prior studies exploring the multifaceted relationships among internet addiction, social anxiety, depression, and NSSI, have resulted in the undervaluation of these factors within the broader mental health ecosystem. This underscores the necessity of the current study.

### Hypotheses

**Figure 1:**
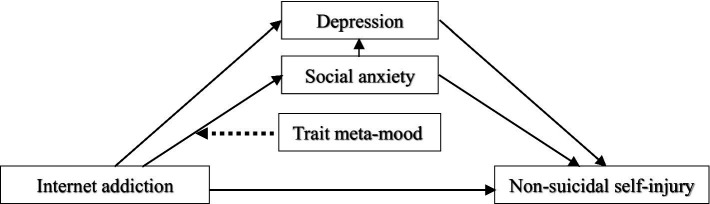
Hypothesis.

In summary, internet addiction, as an increasingly prevalent behavioral issue in modern society, is significantly associated with the occurrence of NSSI among adolescents. Studies indicate that individuals with internet addiction are at greater risk of self-injury and suicide than non-addicted individuals are, with social anxiety and depressive emotions playing important mediating roles in the relationship between internet addiction and NSSI. Furthermore, trait meta-mood, as a personality trait reflecting an individual’s awareness, evaluation, and dynamic regulation of their own emotions, may play a moderating role in the relationships among internet addiction, social anxiety, and NSSI. This is particularly relevant for adolescents, whose underdeveloped prefrontal cortex—responsible for executive control, emotional regulation, and problem solving—exacerbates various issues during this developmental stage. Therefore, to analyze these relationships thoroughly, promote healthy communication patterns and stress-coping abilities among adolescents, reduce internet overuse, and lower the risk of self-harm, this study proposes the hypotheses outlined in Figure 1. The specific hypotheses are as follows:

*Hypothesis 1* Internet addiction has a significantly positive effect on adolescent non-suicidal self-injury.

Internet addiction may lead adolescents to feel isolated and disconnected in real life, thereby increasing the likelihood of non-suicidal self-injury.

*Hypothesis 2* Social anxiety and depression mediate the relationship between internet addiction and non-suicidal self-injury.

Internet addiction triggers social anxiety, which in turn heightens depression. Depression may alter the transmission effect of social anxiety in the relationship between internet addiction and NSSI, further leading to non-suicidal self-injury.

*Hypothesis 3* Trait meta-mood moderates the mediating path between internet addiction and social anxiety.

Trait meta-mood enables individuals to have a “self-aware” understanding of their emotions. This clear self-awareness helps individuals accurately detect emotional fluctuations induced by internet addiction and identify anxiety in interpersonal interactions, thereby preventing self-harm behaviors.

## Methodology

### Participants

The study employed a cross-sectional survey in which onsite data were collected *via* standardized scales. In December 2023, a class-based survey was conducted at three high schools in Heilongjiang Province, China. Prior to the questionnaire, guardians provided written informed consent in a manner compliant with the Helsinki Declaration. The questionnaires were administered under the principles of anonymity, confidentiality, and voluntary participation. A total of 800 questionnaires were distributed, with 716 returned and 692 deemed valid. To ensure adequate statistical power for structural equation modeling (SEM) and mediation effect testing, a priori sample size estimation was performed using G Power 3.1 (Faul et al., 2007). Based on an anticipated medium effect size (*f*^2^ = 0.15), a significance level of *α* = 0.05, and a desired statistical power of 0.95, and accounting for an estimated 20% invalid response rate, the minimum required sample size was calculated to be 580. The obtained valid sample of 692 exceeds this threshold, indicating sufficient statistical power for the proposed analyses. The participants (*N* = 692) ranged in age from 13 to 19 years, with the majority (76.3%) being between 16 and 18 years old. The sample was nearly evenly divided by gender, with 347 males (50.14%) and 345 females (49.86%).

### Measures

#### Internet addiction test (IAT)

The study assessed adolescents’ internet addiction levels *via* the Internet Addiction Test (IAT) developed by Young (Young, 1996). The IAT questionnaire consists of 20 items scored on a 5-point Likert scale: “rarely,” “occasionally,” “sometimes,” “often,” and “always.” Higher scores indicate more pronounced addiction. IAT scores were categorized into normal use (0–30), mild (31–49), moderate (50–79), and severe (80–100) addiction. The IAT has been translated into multiple languages and tested across diverse samples worldwide. It comprises five dimensions: compulsive internet use, withdrawal symptoms and relapse, tolerance, time management issues, and interpersonal and health problems. The scale demonstrated strong reliability, with a Cronbach’s *α* coefficient of 0.928 in the study sample.

#### Deliberate self-harm inventory (DSHI)

The Deliberate Self-Harm Inventory (DSHI) was developed by scholar Gratz and later modified by Lundh et al. for assessing NSSI in adolescents (Lundh et al., 2007). It includes 16 types of non-suicidal self-injury behaviors, such as cutting, burning, scratching, biting, and stabbing, categorized into four major groups: self-laceration, self-poisoning, deliberate recklessness, and self-beating. The scale uses a 4-point scoring system to evaluate the frequency and severity of each non-suicidal self-injury behavior. The frequency of non-suicidal self-injury behavior is measured on a 4-point scale, whereas the severity of physical harm is assessed on a 5-point scale. The total score for non-suicidal self-injury behavior is calculated as the product of the frequency and severity of physical harm. A total score greater than 0 is used as the criterion to determine the presence or absence of non-suicidal self-injury behavior, with a cumulative score greater than 1 indicating the presence of non-suicidal self-injury. Higher scores reflect a higher frequency of self-harm. In this study, the Cronbach’s *α* coefficient for this scale was 0.923.

#### Liebowitz social anxiety scale (LSAS)

The Liebowitz social anxiety scale (LSAS) is a tool widely used internationally to assess the severity of social anxiety disorder and the effectiveness of treatment (Liebowitz, 1987). The self-reported version of the LSAS, adopted in this study, is more suitable for large-scale research and clinical practice. It consists of 24 items, with 11 related to social situations and 13 related to performance situations. The scale evaluates both fear and avoidance symptoms of social anxiety disorder *via* a 4-point scoring system (0 to 3) for each of the two subscales. The total score ranges from 0 to144 and is divided into four factors: social interaction, public speaking, being observed by others, and eating/drinking in public. A higher total score indicates a greater level of social anxiety and fear. In this study, the Cronbach’s *α* coefficient for this scale was 0.979.

#### Beck depression inventory-II (BDI-II)

The beck depression inventory-II (BDI-II) is used to assess the severity of depressive symptoms over the past 2 weeks (Beck et al., 1996). The scale comprises 21 items, with each item scored on a scale ranging from 0 to 3. Each question includes four statements describing varying degrees of depressive mood, and respondents select the option that best reflects their current emotional state, with corresponding scores assigned. The total score ranges from 0 to 63, with higher scores indicating more severe depression. The Chinese version of the BDI-II retains all the original items from the English version and follows the same presentation format. In this study, the Cronbach’s α coefficient for this scale was 0.963.

#### Trait meta-mood scale (TMMS)

The trait meta-mood scale (TMMS-24), a 24-item refinement of the original 48-item scale developed by Spanish researchers (Fernández-Berrocal et al., 2004), assesses meta-emotion through three dimensions: attention (emotional focus), clarity (emotional understanding), and repair (emotional regulation). Widely used internationally (e.g., Japan, South Korea, Germany, China), this version demonstrates high reliability (Cronbach’s α coefficient = 0.923) for evaluating meta-emotion in adolescents.

### Procedure and data analysis

The study selected high school students from a province in northern China as participants, all of whom completed the five aforementioned scales. All data collection and coding procedures were conducted anonymously, with full compliance to ethical standards set by both institutional and national research committees as well as the 1964 Helsinki Declaration and its subsequent amendments. Informed consent was obtained from all participating individuals before their inclusion in the study. IBM SPSS Statistics 22.0 and AMOS 20.0 were used as data analysis tools. SPSS was employed to verify the reliability of the scales and to conduct correlation analyses among the five variables. Structural equation modeling (SEM) was applied *via* AMOS to validate the data, establishing full mediation, partial mediation, and theoretical hypothesis models, followed by model fitting. Hierarchical regression analysis in SPSS was used to test the moderating effect of TMMS.

## Results

### Descriptive statistics and correlation analysis

The study collected data *via* a self-report method. To avoid the impact of common method variance on the relationships between variables, SPSS statistical software was used to test for common method bias. The results indicated that the variance explained by the first unrotated factor was 19.321%, which is below the critical threshold of 40%, suggesting that there was no significant common method bias in this study. Statistical analysis was conducted to perform descriptive statistics and correlation analysis on the five variables: internet addiction, non-suicidal self-injury, social anxiety, Beck Depression, and trait meta-mood.

The descriptive statistics revealed the means, standard deviations, and sample sizes (*N* = 692), reflecting the distribution characteristics of the data, as shown in Table 1. The standard deviations indicate the degree of dispersion within each variable. Non-suicidal self-injury and social anxiety exhibited relatively high standard deviations, suggesting significant variability in these variables among individuals. Depression and trait meta-mood had moderate standard deviations, indicating some variability but not as pronounced as non-suicidal self-injury and social anxiety. The mean and standard deviation of internet addiction were both comparatively low, suggesting relative stability in overall severity while retaining measurable variability among individuals. Based on the established cut-off points of the Internet Addiction Test, participants in this study were classified into the following categories according to their total IAT scores: normal, 30.3% (*N* = 210); mild, 43.1% (*N* = 298); moderate, 24.6% (*N* = 170); severe, 2.0% (*N* = 14).

**Table 1:** Descriptive statistics.

Variables	Mean	SD	*N*
IAT	40.929	15.693	692
NSSI	13.223	20.796	692
LSAS	69.028	36.648	692
BDI-II	15.591	16.111	692
TMMS	64.168	17.716	692

IAT, Internet addiction test; NSSI, Non-suicidal self-injury; LSAS, Liebowitz social anxiety scale; BDI-II, Beck depression inventory-II; TMMS, Trait meta-mood scale. All other tables follow the same format for the annotation.

The correlation analysis results demonstrated significant pairwise relationships among all five variables (*p* < 0.01), confirming Hypothesis H1, as presented in Table 2. Specifically, internet addiction exhibited significant positive correlations with non-suicidal self-injury, social anxiety, and depression, indicating that individuals with higher scores on implicit attitude measures were more likely to display elevated levels of non-suicidal self-injury, social anxiety, and depressive symptoms. Depression was significantly correlated with non-suicidal self-injury (positive), social anxiety (positive), and trait meta-mood (negative). Trait meta-mood was significantly negatively correlated with internet addiction, non-suicidal self-injury, and social anxiety, suggesting that individuals with stronger emotion regulation capabilities tended to score lower on measures of internet addiction, non-suicidal self-injury, and social anxiety.

**Table 2:** Correlation analysis results.

Correlations	IAT	NSSI	LSAS	BDI-II	TMMS
IAT	1				
NSSI	0.278^**^	1			
LSAS	0.301^**^	0.247^**^	1		
BDI-II	0.201^**^	0.217^**^	0.298^**^	1	
TMMS	−0.226^**^	−0.246^**^	−0.290^**^	−0.682^**^	1

**p < 0.01.

### Model selection and path analysis

To verify the relationship between internet addiction and NSSI behavior among adolescents, the structural equation modeling (SEM) method was applied *via* AMOS 20.0 software. A fully mediated model, three partially mediated models, and the theoretical hypothesized model were established and compared. The fit indices of the five models are shown in Table 3. The results indicate that the theoretical hypothesized model has the best fit indices (*χ^2^/df* = 3.253, *RMSEA* = 0. 057, *GFI* = 0. 952, *CFI* = 0. 979, *PCFI* = 0.783), demonstrating the best fit.

**Table 3:** Fit indices of the five models.

Model	Path	*χ*^2^/df	RMSEA	GFI	CFI	TLI
Theoretical hypothesis model	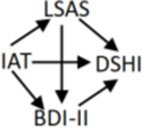	3.253	0.057	0.952	0.979	0.974
Complete mediation model	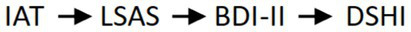	3.794	0.064	0.943	0.973	0.968
Partial mediation model I	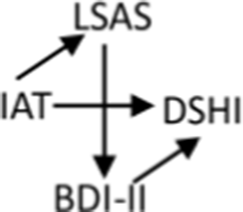	3.393	0.059	0.949	0.978	0.973
Partial mediation model II	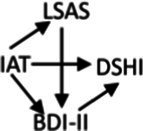	3.338	0.058	0.950	0.978	0.973
Partial mediation model III	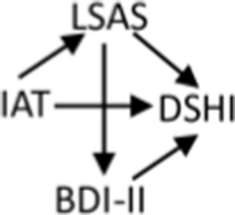	3.313	0.058	0.950	0.979	0.973

**Figure 2:**
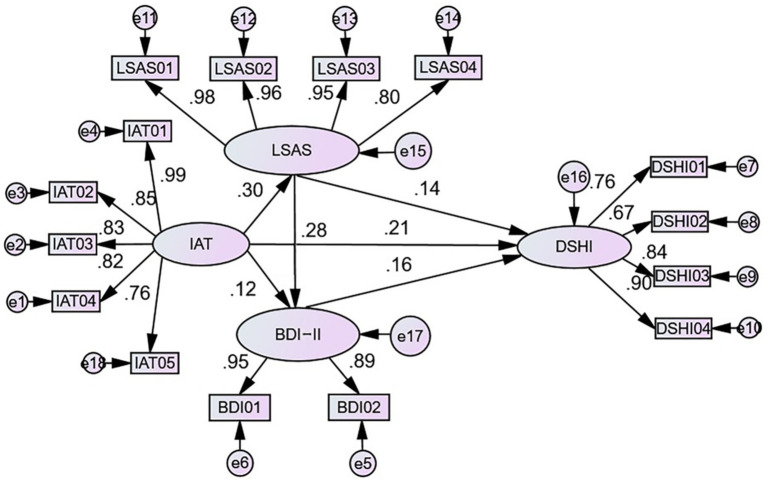
Path analysis diagram of the theoretical hypothesized model.

Next, AMOS was used to calculate the theoretical hypothesized model. The results show that internet addiction has a significant positive effect on social anxiety (standardized path coefficient *γ* = 0.3, *p* < 0.001) and a significant positive effect on depression (γ = 0.12, *p* = 0.003 < 0.01). Internet addiction also has a significant positive effect on non-suicidal self-injury behavior (γ = 0.21, *p* < 0.001), supporting Hypothesis H2. Social anxiety has a significant negative effect on depression (γ = 0.287, *p* < 0.001) and a significant positive effect on non-suicidal self-injury behavior (γ = 0.14, *p* = 0.001). Depression has a significant positive effect on non-suicidal self-injury behavior (γ = 0.16, *p* < 0.001), as shown in Figure 2. In summary, as adolescents’ internet addiction increases, their social anxiety becomes more severe, leading to elevated depression, which in turn increases the likelihood of non-suicidal self-injury behavior.

### Mediation effect test

With a sample size of 2,000 and a confidence level of 95%, the bootstrap method was applied in AMOS to test the mediation effects of the theoretical hypothesized model. After controlling for gender and grade, the results revealed that the direct effect of internet addiction on NSSI behavior was 0.21, accounting for 73.77% of the total effect. The total indirect effect is 0.075, accounting for 26.22% of the total effect. The confidence intervals of the three mediation paths do not include 0, indicating that the mediation effects of all three paths are significant.

Specifically: (1) The internet addiction → social anxiety → NSSI behavior path has an effect value of 0.042, accounting for 14.755% of the total effect, with a relative mediation effect of 56.27%, indicating the highest degree of mediation. (2) The internet addiction → depression → NSSI behavior path has an effect value of 0.0192, accounting for 6.745% of the total effect, with a relative mediation effect of 25.724%, indicating a moderate degree of mediation. (3) The chain mediation effect, i.e., the internet addiction → social anxiety → depression → NSSI behavior path, has an effect value of 0.013, accounting for 4.72% of the total effect, with a relative mediation effect of 18.01%. Table 4 reflects the relationships between the variables in the model and other test parameter values, showing that all paths have significant effects, supporting Hypothesis H2.

**Table 4:** Path analysis and regression estimation results.

Path	Standardized estimate	Unstandardized estimate	Standard error	C.R. (*t* value)	*p*
LSAS ← IAT	0.303	0.825	0.104	7.946	0.000
BDI-II ← IAT	0.118	0.075	0.025	2.941	0.003
BDI-II ← LSAS	0.277	0.064	0.01	6.632	0.000
NSSI ← IAT	0.210	0.208	0.041	5.068	0.000
NSSI ← LSAS	0.137	0.050	0.015	3.250	0.001
NSSI ← BD-II	0.158	0.248	0.066	3.752	0.000

### Moderated mediation effect test

The study used Model 83 from Hayes’ (2012) SPSS PROCESS macro to test whether the relationship between the independent variable (X) and mediator (M) (Path a) was moderated by a moderator (W) while controlling for gender and age. After controlling for gender and age, the moderated mediation model was tested. The results show that after introducing trait meta-mood into the model, the interaction term between internet addiction and trait meta-mood significantly predicts social anxiety (*B* = 0.015, *t* = 3.883, *p* < 0.001). This finding indicates that trait meta-mood moderates the first half of the mediation path where social anxiety mediates the relationship between internet addiction and NSSI behavior, as shown in Table 5.

**Table 5:** Test of the moderated mediation model.

Constant	LSAS					
	coeff.	se	*t*	*p*	LLCI	ULCI
IAT	0.701	0.089	7.847	0.000	0.525	0.876
TMMS	−0.521	0.075	−6.974	0.000	−0.668	−0.375
IAT*TMMS	0.015	0.004	3.883	0.000	0.008	0.023
*R*	0.401					
*R-sq*	0.161					
*F(df)*	44.029(3^***^)					

***p < 0.001.

Further analysis reveals that at three levels of trait meta-mood, the mediating effect of internet addiction on social anxiety gradually increases as trait meta-mood increases from a lower level (M—1SD) to an average level (M) and a higher level (M + 1SD), as shown in Table 6. For participants with lower trait meta-mood (M—1SD), internet addiction has a significant positive predictive effect on social anxiety, with a mediating effect value of 0.036 and a 95% bootstrap confidence interval of (0.013, 0.065), excluding 0. For participants with average trait meta-mood (M), the mediation effect increases to 0.058, with a 95% bootstrap confidence interval of (0.026, 0.095), also indicating a significant mediation effect. For participants with higher trait meta-mood (M + 1SD), internet addiction positively predicts social anxiety, with the mediating effect value increasing to 0.08, the highest among the three levels. Contrary to Hypothesis H3, which posits that trait meta-mood influences individual emotions and partially blocks self-injury behavior, the results show that trait meta-mood enhances the mediation effect, thereby intensifying social anxiety, depression, and non-suicidal self-injury behavior. Furthermore, as shown in Table 6, the indirect effect of internet addiction on NSSI through the chain mediation of social anxiety and depression also exhibited a gradient change across different levels of trait meta-mood (*eff1* = 0.008, *eff2* = 0.014, and *eff3* = 0.018). This indicates that trait meta-mood moderates not only the first half of the mediation pathway but also the entire chain-mediation process.

**Table 6:** Mediation effects at different levels of trait meta-mood.

Path	TMMS	Effect	BootSE	BootLLCI	BootULCI
IAT → LSAS → NSSI	*eff1(M-1SD)*	0.036	0.013	0.013	0.065
	*eff2(M)*	0.058	0.018	0.026	0.095
	*eff3(M + 1SD)*	0.08	0.024	0.036	0.132
	*eff1(M-1SD)*	0.008	0.004	0.002	0.018
IAT → LSAS → BDI-II → NSSI	*eff2(M)*	0.014	0.006	0.003	0.027
	*eff3(M + 1SD)*	0.018	0.008	0.004	0.038

## Discussion

This study explored the relationship between internet addiction and NSSI behavior among adolescents, examining the roles of social anxiety, depression, and trait meta-mood. The findings revealed that internet addiction is a risk factor for NSSI behavior among Chinese adolescents, which is consistent with the findings of previous research. Internet addiction not only directly and positively predicts NSSI behavior but also indirectly influences it through the chain mediation of social anxiety and depression. This discovery is the first to validate the importance of the progressive mediation pathway (social anxiety → depression) in this relationship. Trait meta-mood moderates the mediation model by regulating the first half of the mediation pathway, thereby enhancing the mediating effect of social anxiety. The indirect effect values increase as the level of trait meta-mood increases, indicating its moderating role in the multistage mediation process. However, the study did not find the expected protective effect of trait meta-mood on individuals; instead, it confirmed that higher levels of trait meta-mood exacerbate the transformation of risk factors into self-injury behavior. This result revises the theoretical assumption that trait meta-mood solely functions as a protective trait, highlighting its complexity in different psychological mechanisms. The findings of this study are discussed below.

### Relationship between internet addiction and non-suicidal self-injury behavior in adolescents

The study revealed that internet addiction is a risk factor for NSSI behavior among Chinese adolescents. This conclusion supports the notion that the internet can influence self-harm, as confirmed by cross-sectional studies across different national groups (Kim et al., 2023). Longitudinal studies using cross-lagged panel models have also shown that individuals often encounter NSSI-related content online before resorting to violent self-harm methods (Dunlop et al., 2011). Exposure to graphic online materials can trigger impulses and behaviors related to self-injury (Lewis and Baker, 2011), influencing how individuals express or describe their self-harm. Neurophysiological research indicates that, compared with healthy controls, internet addicts exhibit specific functional changes in brain regions associated with conflict monitoring, reward processing, and cognitive control, predisposing them to make riskier decisions (Seok et al., 2015). In particular, individuals with internet gaming disorder show reduced gray matter volume in brain regions related to inhibitory control (Yao et al., 2017), providing a biological basis for the association between internet addiction and NSSI behavior in adolescents. From another perspective, non-suicidal self-injury has been classified as a behavioral or process addiction, which shares neurobiological and psychological mechanisms with addiction, such as dysregulation of the endogenous opioid system (Blasco-Fontecilla et al., 2016). When self-injury is viewed as an addiction, individuals may experience more frequent and severe self-harm, unintended serious injuries, and suicidal ideation. In such cases, the individual’s self-injury experiences gradually become normalized.

However, some studies have reported no clear association between internet addiction and NSSI behavior. This inconsistency in results may be attributed to differences in population characteristics and research methodologies. In addition to the study design, the use of various measurement tools and criteria to assess the prevalence of self-harm and internet addiction can also lead to divergent findings. Studies that fail to distinguish between non-suicidal self-injury and broader self-harm behaviors may overestimate the association between internet addiction and NSSI. The variability in the relationship between internet addiction and NSSI highlights the need to contextually consider relevant factors and explore the underlying mechanisms to uncover more accurate and scientific associations. This study incorporates social anxiety and depression as key variables to gain a deeper understanding of the complex relationship between internet addiction and NSSI behavior.

### Chain mediating role of social anxiety and depression

Neuropsychological issues play a significant role in explaining risk behaviors among adolescents with internet addiction, with the increased prevalence of depression, anxiety, and other mental health problems being key factors. According to the syndrome model of addiction, psychosocial factors are considered indispensable components in explaining addictive behaviors. As one of the psychosocial factors explored in the internal mechanisms of the relationship between internet addiction and NSSI, social anxiety was analyzed as a mediating factor. The findings indicate that high levels of social anxiety amplify the effect of internet addiction on NSSI, producing a stronger indirect effect. Specifically, internet-addicted individuals, owing to their excessive immersion in the virtual online world, have become increasingly disconnected from real-world social interactions, leading to heightened social withdrawal and an elevated risk of social anxiety disorder. Anxiety plays a driving role in increasing the risk of suicidal ideation, making adolescents more vulnerable when facing reality. Previous research supports this finding, explaining the mediating role of social anxiety between NSSI and internet addiction and revealing the potential mechanism through which internet addiction indirectly influences NSSI by exacerbating social anxiety. On the one hand, adolescents, who generally have strong self-esteem, are particularly concerned with others’ opinions and evaluations of them. Individuals with high anxiety, due to low self-esteem and high avoidance tendencies, are more prone to self-harm (Tariq et al., 2021). On the other hand, social media provides a comfortable virtual environment for individuals with social anxiety, but this virtual environment differs from the real world. Therefore, excessive use of social media may replace real-world activities, weaken offline social support systems, and exacerbate symptoms of social anxiety, leading to destructive behaviors (Wu et al., 2024).

Further analysis revealed that depression, as a negative emotion, mediates the relationship between internet addiction and NSSI, with one of the primary motivations for engaging in NSSI being the need to cope with adverse emotions. In particular, the relief experienced following self-injury may be especially pronounced, making it difficult to stop the behavior, especially when numerous underlying factors—such as intense distress, depression, and anxiety—persist and when the individual lacks alternative coping mechanisms. Thus, although many individuals may feel shame or anger when considering the consequences of self-harm, they may continue their behavior. The perceived benefits of continued self-harm may outweigh the benefits of stopping, and individuals may not know or believe in other ways to cope with their pain or other factors driving self-harm (Pritchard et al., 2021). If individuals are repeatedly exposed to messages suggesting that stopping self-harm is difficult (if not impossible) or if these messages are inherently filled with hopelessness and despair, self-harm may become normalized or reinforced (Lewis and Seko, 2016).

Social anxiety and depression play a chain mediating role in the relationship between internet addiction and NSSI behavior. Numerous previous studies on anxiety and depression have indicated their interconnectedness. In empirical research, Jacobson et al.’s longitudinal investigation into the relationship between early adolescence anxiety and depression revealed that anxiety predicts subsequent depression. Individuals with social anxiety are more likely to develop depression, especially when they become immersed in the virtual online world and spend less time engaging in social or group activities with family or peers, exacerbating the manifestation of problematic behaviors (Jacobson and Newman, 2014). Psychologists at the University of Washington reported, while examining the overlap between symptoms of these two conditions, that directly addressing related psychological issues can alleviate both social anxiety and depressive emotions (Langer et al., 2019). For the adolescents in this study, spending excessive time online inevitably deprives them of social interactions, which are crucial sources of personal growth. With the reduction in social reinforcement resources, these individuals are more prone to depression and adopt coping strategies suited to a depressive state: adaptive coping, strategies that reduce stress levels, and maladaptive coping, strategies that increase stress levels. Notably, internet use can, in some cases, develop into a maladaptive coping strategy, inducing symptoms of psychopathology or self-injury due to online experiences (Kaess et al., 2014). These findings suggest that treating mental disorders at an early stage, such as through emotional management strategies, can delay or prevent subsequent suicidal behaviors. For this reason, the study analyzed the moderating role of trait meta-mood in the mediation model.

### The moderating role of trait meta-mood

The moderating role of trait meta-mood is reflected primarily in its potential impact on NSSI behavior among adolescents, particularly in how it influences internet addiction and social anxiety. Individuals with high trait meta-mood may be more prone to emotional exhaustion, excessively analyzing the sources of their emotions without effectively regulating them. This emotional sensitivity trait may amplify adolescents’ cognitive biases toward stress, exacerbating the risks of social anxiety and internet addiction and thereby increasing the likelihood of self-injury. Specifically, internet addicts who engage in excessive and maladaptive internet use are more common among emotionally unstable and cognitively impulsive individuals with low emotional competence. However, individuals with high levels of trait meta-mood exhibit similar behavioral tendencies, as those with heightened emotional sensitivity are prone to emotional rumination, leading to excessive anxiety in real-world social situations and reduced resistance to negative behaviors (Fernández-Martínez et al., 2023). Therefore, we propose that trait meta-mood exerts a negative moderating effect by influencing individuals’ emotional experiences. This study confirms the moderating role of trait meta-mood in the mediation model of internet addiction and NSSI. Individuals with high trait meta-mood are often trapped in emotional cognitive overload, making them more likely to adopt avoidant coping strategies, thereby reinforcing the risks of social anxiety and internet addiction and increasing the occurrence of NSSI.

Notably, while the results in Table 2 suggest that higher trait meta-mood may generally serve a protective role, Table 6 reveals a contradictory pattern: among individuals with elevated trait meta-mood, the indirect effect of internet addiction on social anxiety is stronger. Although these two findings may appear inconsistent on the surface, they reflect the dual nature of trait meta-mood (Gohm and Clore, 2002). Trait meta-mood is a multidimensional construct comprising emotional awareness, clarity, and repair. Although heightened emotional awareness is usually considered protective, our findings indicate that an overall increase in trait meta-mood, particularly when accompanied by increased emotional attention without effective regulation, may enhance adolescents’ tendency toward emotional monitoring or rumination. On the one hand, improved emotional awareness and understanding can help individuals recognize emotional fluctuations (Gohm and Clore, 2000). On the other hand, in the absence of adaptive regulatory strategies, individuals may engage in emotional rumination, which can amplify anxious experiences and thereby exacerbate the negative psychological impact of internet addiction (Eckland and Berenbaum, 2021). This suggests that the role of trait meta-mood is not unidirectional, its specific function in psychological mechanisms depends on the interplay of individual emotion regulation strategies and coping styles, as well as the balance between emotional awareness and adaptive emotion regulation capacity.

Moreover, some scholars have studied trait meta-mood as a personality trait, linking it closely to addictive behaviors and considering it a significant predictor of social anxiety. Individuals with high scores for this trait are more likely to over reflect on real-world social interactions, leading them to immerse themselves in controllable virtual environments (Khoshakhlagh and Faramarzi, 2012). The use of the internet provides a platform for individuals to repeatedly validate negative emotions, and those with high trait meta-mood are more likely to adopt maladaptive strategies such as emotional rumination, creating a vicious cycle of “emotional analysis-anxiety amplification-internet addiction” (Verissimo, 2005). This, in turn, exacerbates social anxiety, driving them to become addicted to online activities that offer instant gratification. This perspective further explains the moderating role of trait meta-mood in the relationship between internet addiction and social anxiety. Individuals with high trait meta-mood develop cognitive rigidity due to their excessive pursuit of “correct emotional analysis,” which focuses their emotional regulation strategies on introspection rather than action. These traits collectively contribute to the associations between emotional regulation sensitivity and social anxiety or problematic internet use (Sertbas et al., 2020). Additionally, trait meta-mood has a compensatory effect; when individuals excessively rely on emotional regulation rather than behavioral regulation, their emotional sensitivity traits weaken their adaptability to stressful events, impairing social functioning and increasing anxiety (Bonanno, 2004). This suggests that trait meta-mood has a potential aggravating effect on moderating the relationship between internet addiction and social anxiety (Quaglieri et al., 2021). Therefore, when designing prevention and educational programs targeting internet addiction, it is essential to consider the role of trait meta-mood. Care must be taken to avoid emotional training that may inadvertently increase emotional fixation tendencies in adolescents. Instead, efforts should focus on guiding them to establish adaptive behavioral regulation patterns to reduce the occurrence of NSSI behavior.

## Theoretical and practical implications

### Theoretical implications

The study’s innovation lies in breaking through the traditional single-path analysis framework and constructing a chain mediation model of Internet Addiction → Social Anxiety → Depression → Non-suicidal Self-Injury, providing a new theoretical framework for understanding the comorbidity mechanisms of addictive behaviors and self-injury among adolescents. By incorporating trait meta-mood into the explanatory system of addiction and self-injury behaviors, this study addresses the previous lack of attention given to dynamic emotional regulatory mechanisms. Integrating neurophysiological evidence with psychosocial mechanisms clarifies how individual psychological resources buffer risk behaviors, deepening the theoretical integration of the bio-psycho-social model in the field of adolescent problem behaviors. This provides a new direction for the future exploration of addiction and self-injury behaviors.

### Practical implications

This study is the first to introduce trait meta-mood as a key factor, offering dual cognitive and behavioral targets for adolescent mental health interventions. From a cognitive perspective, this study emphasizes the importance of early intervention in internet use behaviors while also highlighting the need to address adolescent emotional issues to prevent the psychological fixation generated by emotional sensitivity and excessive monitoring mechanisms, which can exacerbate the transmission of negative emotions and behaviors. This finding has significant implications for school mental health education; schools should develop modular curricula that integrate internet behavior management and emotional skills training. This study also provides new goals for clinical interventions, suggesting the inclusion of meta-emotion awareness training modules in cognitive behavioral therapy to reduce the psychological exhaustion caused by excessive emotional monitoring in adolescents. Furthermore, it offers valuable insights for public health policy development, advocating for the establishment of a comprehensive intervention system that combines technology monitoring and psychological resilience cultivation to address adolescent internet use, providing a scientific basis for preventing mental health crises in the digital age.

### Limitations

Differences in research methods and participant baseline conditions may influence the study’s results. Cross-sectional studies cannot directly confirm causal relationships between internet addiction and NSSI, and whether their relationship is unidirectional or bidirectional remains to be explored. The study’s data were obtained through self-reports from participants, which may introduce potential issues such as underreporting or biased recall. While demographic factors were controlled for in the analysis of variable relationships, potential confounding factors could not be fully adjusted for, which may lead to overestimation or underestimation of the strength of these associations.

### Future directions

Future research could adopt experimental methods to more clearly demonstrate causal relationships between variables by manipulating independent variables. To increase the validity of related findings, longitudinal studies involving long-term follow-up or cross-lagged panel designs could be employed to explore the stress perception process comprehensively and identify critical turning points in the conditioning process.

## Conclusion

The study revealed that internet addiction is a significant risk factor for NSSI behavior among adolescents. Internet addiction not only directly increases the risk of NSSI but also indirectly influences it by exacerbating social anxiety, which acts as a mediating variable. Depression also plays a crucial progressive role in this process, with the presence of depressive symptoms increasing the complexity of the combined pathway of social anxiety and internet addiction on NSSI behavior.

Higher levels of trait meta-mood strengthen the mediating effect of social anxiety and depression on the relationship between internet addiction and NSSI. Individuals with high emotional awareness and understanding abilities, if they lack adaptive regulation strategies, may experience cognitive rigidity due to excessive focus on emotions, leading to the accumulation of negative emotions and increasing the probability of risk behaviors. This highlights the importance of fostering adolescents’ mental health by developing their emotional competence on the basis of individual traits to help them better cope with various psychological challenges.

## Data Availability

The raw data supporting the conclusions of this article will be made available by the authors, without undue reservation.
